# Side-Effects of Public Health Policies Against Covid-19: The Story of an Over-Reaction

**DOI:** 10.3389/fpubh.2021.696818

**Published:** 2021-09-13

**Authors:** Edouard Lansiaux, Noé Tchagaspanian, Juliette Arnaud, Pierre Durand, Mark Changizi, Joachim Forget

**Affiliations:** ^1^Global Variations, Genève, Switzerland; ^2^Ecole Normale Supérieure, Paris, France; ^3^Human Factory, Columbus, OH, United States; ^4^Assemblée Nationale, Paris, France

**Keywords:** COVID-19, lockdown, curfew, mask, side-effects, health policy, public health, non-pharmaceutical intervention

## Introduction

The world has been facing a coronavirus disease (COVID-19) pandemic since November 2019. While there may have been a short period at the start when the risk of the pandemic was underestimated, by February and March of 2020, the Western world reacted in earnest with varieties of non-pharmaceutical interventions. However, the overall effects of those interventions had not at that point been sufficiently studied, and in many cases what existing literature existed did not recommend them. Furthermore, beyond the issue of whether the interventions were narrowly effective, there is the issue of their potential side effects on global health, something that was given surprisingly little attention.

The medical motto “*primum non nocere*” (≪*first, do not harm*≫), a moral principle everyone should at least consider following, was evidently not observed. The potential up sides of the interventions were promoted and communicated, but rarely the myriad possible down sides. This opinion article highlights a variety of the down sides in an effort to emphasize the broad range of complex issues that must be balanced when governments enact policy.

## Lockdowns

### Epidemiological Effects

In the Middle Ages, before the discovery of pathogen vectors, patients were seen as presenting a health and social risk. Since then, “detect, isolate, treat” has almost always been, and still is, the *credo*. Isolation used to be *selective*. For example, there were *lazarettos* that were used to keep ship passengers or patients in quarantine (1). In seventeenth century London, only infected families were “shut-up” in their homes, their doors being marked with red crosses (2) in order to prevent other people from paying them visits. A general lockdown extending to healthy or asymptomatic people was very uncommon, almost without historical precedent, and lacking scientific basis.

Stay-at-home mandates' impact on mortality is subject to debate, for while some studies report its epidemiological impact (3), many others suggest an absence of COVID-19 mortality reduction due to the lockdown (4, 5). Moreover, the comparison of pre- and post- lockdown observations reveals a counter-intuitive slowdown in the decay of the epidemic *after* lockdown (6, 7). In a nutshell, many studies now suggest lockdown inefficacy for COVID-19 mortality, and even sometimes SARS-CoV-2 mere transmission (8, 9). A Stanford epidemiological study (10) did not find significant benefits on case growth of more restrictive non-pharmaceutical interventions (NPIs).

More important for our purposes here, though, are the side effects, and epidemiologically there is considerable evidence now of significant increased mortality due to lockdowns and the connected changes to medical practice that occurred during the pandemic. For example, according to a Centers for Disease Control and prevention (CDC) report (11) concerning excess deaths in the US between January 26th 2020 and October 3rd 2020, 1/3 of them (or 100,000) were not COVID-19-related (12). It is beyond the scope of this short piece to review the literature on these lockdown-related deaths, but it is crucial to note that the important comparison is not the number of lockdown-related deaths to the number of COVID-19-related deaths, but, rather, the number of lockdown-related deaths to the number of COVID-19 deaths *averted* by virtue of the interventions.

According to a study conducted by the National Bureau of Economic Research (13), for the overall US population, the proportion of COVID-19 related unemployment is today between two and five times larger than the typical unemployment shock, resulting in a 3.0% increase in mortality rate and a 0.5% drop in life expectancy over the next 15 years. Deaths from drug and alcohol misuse also significantly increased during the lockdown period in comparison to the same period in 2018 (14). The damage to the economy by lockdowns will cost many years of life—and poverty is a silent killer (15).

Lockdowns are far from being a magic spell that can save the world from a pandemic: They might not even narrowly work to lower mortality, and appear to lead to their own share of non-COVID-19 deaths (16).

### Psychological Side-Effects

During this COVID-19 period, economic vulnerability was associated with a strong risk of stress and worsening mental health (17). According to Sonia Mukhtar, lockdowns, whose consequences are self-isolation quarantine and social distancing, constituted collective traumatic events that are perceived by people as serious threats, and have already resulted in a considerable loss of life and in an impoverishment of global hygiene (18). Indeed, as Mingke Song assessed for China, COVID-19 and lockdown policies not only brought upon a life crisis, but also incurred psychological stress: tension, anxiety, fear and despair among affected populations (19). A review also found that some factors increasing women's vulnerabilities to violence have been exacerbated during the lockdown period (20).

The psychological effects of isolation in non-epidemic situations have already been studied in specific cases, such as that of imprisonment (21, 22). Not everyone is able to be as positive and creative as Xavier de Maistre was when he wrote his impressive *Voyage autour de ma chambre* during his imprisonment in Turin, in 1794.

Previous epidemics and the specific lockdowns they caused also had psychological effects, and were described by specialists (23, 24). The risk of PTSD (post-traumatic stress disorder) symptoms is at its highest, even after some time, and even after home quarantine.

Lockdowns led to most medical care being done via cyber-visits, which greatly reduces the physician's ability to perceive health signs. Doctors are often not even consciously aware of their fine-tuned perceptual abilities. For example, our variety of color vision evolved so as to sense oxygenation modulations under the skin (for recognition of emotion, health and state) (25), and it has been recognized since the Greeks that the acute pallor of the skin is helpful for diagnosis (26). These blood-mediated health signals are only visible in person, not through cameras.

### Physiological Effects

Lockdowns also increase the duration of time for which people are sedentary, which has a variety of harmful side effects including: altered energy expenditure, adipogenic signaling, immunomodulation, autonomic stability, and hormonal dysregulation perpetuating underlying chronic diseases such as obesity, cardiovascular disease, cancer and mental health disorders, which are grave physiological effects (27). In addition, Digital Eye Syndrome (user's visual system regulation difficulty mainly caused by an overuse of digital devices) may have been exacerbated precisely because of lockdowns (28).

## Masks

### Effectiveness

The debate regarding the effectiveness of masks is still ongoing. Indeed, some believe masks are ineffective (for both this coronavirus virus and influenza variants) (29–31), others defend the simple surgical mask efficiency (this is the most common scientific opinion), and others are calling for more effective masks (32).

Even supposing face masks might provide some measure of protection, there are side effects that could undermine any efficacy they may have. First, wearing a mask may give a false sense of security and make people less compliant with social distancing, ventilation and other important infection control schemes (33, 34). Second, people have to avoid touching their masks and adopt other management measures, otherwise masks may be counterproductive (35).

While face masks can stop larger droplets, such droplets tend to fall to the ground due to their weight (36–38), and are not the route for viral transmission. Viruses spread via smoke-like aerosols (39) via breath (or flatulence), which go through and jet out the sides of surgical masks, and infect mainly by inhalation deep into the lungs. Despite the risk of inhaling/exhaling infected virions via leaks of particles, this was never evaluated in applied norms for surgical masks, and only for Personal Protective Equipment (PPE) under the Filtering Facepiece Particles (FFP) norm in Europe, and N (e.g., N95) in the USA. Moreover, the European norm for surgical masks (EN14683) as well as the US (ASTM) only applies to Bacterial Filtration Efficiency (BFE), and the size of the bacteria used for testing (3 microns) is much larger than the SARS-CoV-2 [maximum size of 140 nm (40)]. Virus filtration efficiency (VFE) was never tested in Chinese and European norms.

### Psychomotor Effects

Mask-wearing could affect infants' and children's psychomotor development, as well as facial recognition (41). The still-face effect (42), for example, illustrates the human grasp of emotional expressions from very early in life, something obviously interrupted in a world filled with masked people.

Moreover, one could speculate that because brain areas in the left fusiform cortex were recycled for reading expertise (43), while face recognition expertise is more lateralized in the homolateral fusiform cortex (44), some upcoming dyslexic syndromes could be expected as a consequence of the lack of face visual recognition skills' development due to bilateral ventral stream impairment.

Masks also block vision of one's lower far peripheral visual field, which is crucial for visuomotor feedback when engaged in walking (45–48), something almost never consciously realized (49). Falls are a major public health concern because falls are the second leading cause of accidental or unintentional injury deaths worldwide—each year approximately 650,000 individuals die from falls (50).

### Psychological Effects

Masks severely handicap us in our most fundamental way of communicating—our emotional expressions (51–53), something that is as relevant in health diagnoses (54) as it is in regular life. For instance, a randomized clinical trial has shown that health care professionals wearing masks have a significant and negative impact on the patient's perceived empathy and diminish positive effects of relational continuity (55).

A recent study also showed that each type of mask caused a low-pass filter effect, attenuating higher frequencies (2,000–7,000 Hz) in the speaker's voice by 3–4 dB (medical mask) and nearly 12 dB for the N95 mask (respirator/FFP) (56). In addition to this, masks significantly prevent binding mechanisms through which de-synchronized auditory and motor signals from language are usually fused into conscious workspace—a phenomenon known as the McGurk effect (57).

Also, a review notably supports the idea that panic-prone individuals may be at higher risk of respiratory discomfort when wearing RPDs, thereby reducing their tolerance for these devices (58).

### Dermatological Effects

Many studies have described the dermatological impact of prolonged mask wearing. Mask wearing induces itches (59) and contact dermatitis (60). The most common adverse skin reactions among healthcare workers wearing N95 masks have been nasal bridge scarring (68.9%) and facial itching (27.9%) (61), nasal bridge, cheeks and chin (35.5%) (62). N95 respirators are associated with more skin reactions than medical masks (63), and skin tears and open wounds such as these are a themselves potential source of infection (64). Last but not least, the current form of fluid resistant surgical masks (FRSM) used in day-to-day practice has elastic ties that go behind the ears, and an extended use of these masks causes discomfort and irritation behind them, especially if they are used for prolonged procedures (65).

### Physiological Effects

This first randomized cross-over study concerning the effects of surgical masks and FFP2/N95 masks on cardiopulmonary exercise capacity yields clear results: both varieties of mask have a marked negative impact on exercise parameters (66). Furthermore, a German MD thesis (67) showed that the usage of a face mask leads to: increased rebreathing of expelled carbon dioxide; significant increased respiratory rate and hyperventilation; increased heart rate; increase in CO_2_ in the blood; hypoxemia, which is an abnormal decrease in the partial pressure of oxygen in the arterial blood; hypercapnia, which is an increase in the pressure of CO_2_ in the blood. To sum up, as WHO claimed in August 2020: “People should not wear masks when exercising, as masks may reduce the ability to breathe comfortably” (68).

A final consequence of universal mask wearing worth mentioning is one at the societal level: once an unmasked face becomes verboten in most public circumstances, it can end up psychologically treated as a “private part” that must be covered, like all our private parts, something that can be difficult to undo.

## Conclusion

Our opinion article highlighted just some of the many side effects of NPIs that have been adopted by our governments since the COVID-19 crisis began. Even in a terrible epidemic, decisions cannot be taken without an exhaustive risk-benefit analysis, not to mention consideration of civil liberties.

Other potential directions for government responses include policies encouraging better ventilated indoor spaces, “test, trace, isolate” (on a specific scale and not a globalized one) as it was applied in Asian countries, mass vaccinations, and early treatments [although none is actually proven as effective yet, such as the ACEi/ARBs example (69)].

The responses of governments need to be guided by scientific decision-making algorithm (70) and when this dialogue between public authorities and scientists exist, it allows superior pandemic management (71).

## Author Contributions

MC and JF designed the research. EL and NT conducted the research and wrote the first draft of the manuscript. EL, NT, JA, PD, MC, and JF contributed to the writing of the manuscript. All authors contributed to the data interpretation, revised each draft for important intellectual content, and read and approved the final manuscript.

## Conflict of Interest

The authors declare that the research was conducted in the absence of any commercial or financial relationships that could be construed as a potential conflict of interest.

## Publisher's Note

All claims expressed in this article are solely those of the authors and do not necessarily represent those of their affiliated organizations, or those of the publisher, the editors and the reviewers. Any product that may be evaluated in this article, or claim that may be made by its manufacturer, is not guaranteed or endorsed by the publisher.
